# Outbreak of Highly Pathogenic Avian Influenza A(H5N1) Viruses in U.S. Dairy Cattle and Detection of Two Human Cases — United States, 2024

**DOI:** 10.15585/mmwr.mm7321e1

**Published:** 2024-05-30

**Authors:** Shikha Garg, Carrie Reed, C. Todd Davis, Timothy M. Uyeki, Casey Barton Behravesh, Krista Kniss, Alicia Budd, Matthew Biggerstaff, Jennifer Adjemian, John R. Barnes, Marie K. Kirby, Colin Basler, Christine M. Szablewski, Malia Richmond-Crum, Erin Burns, Brandi Limbago, Demetre C. Daskalakis, Kimberly Armstrong, David Boucher, Tom T. Shimabukuro, Michael A. Jhung, Sonja J. Olsen, Vivien Dugan

**Affiliations:** ^1^Influenza Division, National Center for Immunization and Respiratory Diseases, CDC; ^2^One Health Office, National Center for Emerging and Zoonotic Infectious Diseases, CDC; ^3^Office of Public Health Data, Surveillance, and Technology, CDC; ^4^National Center for Immunization and Respiratory Diseases, CDC; ^5^Administration for Strategic Preparedness and Response, Washington, DC.

**Summary**

**What is already known about this topic?**

Influenza A(H5) virus infection was detected in two U.S. farm workers during a multistate outbreak of A(H5N1) viruses in dairy cows; these are the first known instances of presumed cow-to-human transmission of avian influenza A viruses.

**What is added by this report?**

Approximately 350 exposed farm workers are being monitored; one of the two cases was identified via daily, active monitoring. Surveillance has identified no unusual influenza activity trends in the United States. A(H5) candidate vaccine viruses are available, and laboratory analyses indicate that A(H5N1) viruses circulating in cows and other animals are susceptible to FDA-approved antivirals.

**What are the implications for public health practice?**

Current risk to the U.S. public from A(H5N1) viruses is low; however, persons exposed to infected animals or contaminated materials, including raw cow’s milk, are at higher risk and should take precautions and self-monitor for illness. A One Health (human, animal, and environmental) approach is critical to preparing for circumstances that could increase risk to human health.

## Abstract

On April 1, 2024, the Texas Department of State Health Services reported that a dairy farm worker had tested positive for highly pathogenic avian influenza A(H5N1) virus after exposure to presumably infected dairy cattle; CDC confirmed these laboratory findings. A(H5N1) viruses were found in high concentrations in unpasteurized (raw) milk from infected cows. CDC is collaborating with the U.S. Department of Agriculture, the Food and Drug Administration, the Administration for Strategic Preparedness and Response, the Health Resources and Services Administration, the National Institute of Allergy and Infectious Diseases, and state and local public health and animal health officials using a coordinated One Health approach to identify and prepare for developments that could increase the risk to human health. Activities include monitoring of exposed persons, conducting syndromic and laboratory surveillance, planning epidemiologic investigations, and evaluating medical countermeasures. As of May 22, 2024, approximately 350 farm workers with exposure to dairy cattle or infected raw cow’s milk had been monitored. These monitoring efforts identified a second human A(H5) case with conjunctivitis in Michigan, which was reported on May 22, 2024. CDC considers the current risk to the U.S. public from A(H5N1) viruses to be low; however, persons with exposure to infected animals or contaminated materials, including raw cow’s milk, are at higher risk for A(H5N1) virus infection and should take recommended precautions, including using recommended personal protective equipment, self-monitoring for illness symptoms, and, if they are symptomatic, seeking prompt medical evaluation for influenza testing and antiviral treatment if indicated. Pasteurization inactivates A(H5N1) viruses, and the commercial milk supply is safe for consumption; however, all persons should avoid consuming raw milk or products produced from raw milk. Importantly, the risk to the public might change based on whether A(H5N1) viruses acquire genetic changes that increase their transmissibility to and among humans, which could increase the risk of an influenza pandemic.

## Investigation and Findings

### Identification of Two Human Cases of Influenza A(H5) Virus Infection

On April 1, 2024, the Texas Department of State Health Services reported, after confirmation by CDC, that a commercial dairy farm worker tested positive by real-time reverse transcription–polymerase chain reaction (RT-PCR) for highly pathogenic avian influenza (HPAI) A(H5N1) virus infection after exposure to dairy cattle presumed to be infected with A(H5N1) viruses*^,^^†^; CDC confirmed laboratory findings through RT-PCR and sequencing (*1*). The patient only experienced conjunctivitis without other signs or symptoms, was instructed to isolate, was treated with oseltamivir, and recovered. No illness was identified among the patient’s household members, all of whom received oseltamivir postexposure prophylaxis. One week earlier, the U.S. Department of Agriculture had reported a multistate outbreak of A(H5N1) viruses in dairy cows.^§^ A(H5N1) viruses were also detected in barn cats, birds, and other animals (e.g., one raccoon and two opossums) that lived in and around human habitations and that died on affected farms.^¶^ Genetic sequencing of the A(H5N1) virus from infected cattle and the farm worker** identified clade 2.3.4.4b; this clade has been detected in U.S. wild birds, commercial poultry, backyard flocks, and other animals since January 2022 (*2*). On May 22, 2024, the Michigan Department of Health and Human Services reported an A(H5) case in a dairy farm worker on a farm confirmed to have A(H5N1) virus in cattle; this person was enrolled in an active text-based monitoring program and reported only eye symptoms.^††^ The investigation into this second case is ongoing. These two cases are the first known instances of presumed cow-to-human spread of an avian influenza A virus.

### Influenza A(H5N1) Viruses in U.S. Dairy Cattle

Although first reported in March 2024, A(H5N1) virus infection of U.S. dairy cows might have been occurring since December 2023, according to preliminary data (*3*). As of May 22, 2024, infected dairy cows had been identified in 52 dairy cattle herds in nine states^§§^ (Colorado, Idaho, Kansas, Michigan, New Mexico, North Carolina, Ohio, South Dakota, and Texas). Signs in cattle were nonspecific and included decreased milk production, reduced rumination, and thickened (colostrum-like) milk consistency; some cows also had clear nasal discharge. High A(H5N1) virus levels have also been found in unpasteurized (raw) milk from infected cows(*4*).

### Human Cases of Influenza A(H5N1) Worldwide

From 1997 through late April 2024, a total of 909 sporadic human A(H5N1) cases were reported worldwide from 23 countries; 52% of human cases have been fatal (*2*); of the 909 cases, 26 human A(H5N1) cases have been reported from eight countries, including seven deaths, since 2022. Since these numbers were last updated, two additional human A(H5) cases have been detected including the case from Michigan and one case in Australia. Nearly all reported human A(H5N1) cases had reported recent exposure to poultry. In the United States, three human A(H5) cases have been identified to date; all patients had mild illness, were not hospitalized, and fully recovered. The first occurred in April 2022 in a person from Colorado with direct exposure to infected poultry, who only reported fatigue,^¶¶^ and the second and third occurred in dairy farm workers with conjunctivitis referenced in this report.

### U.S. Outbreak Response Activities

Activities implemented using a One Health*** approach to respond to this outbreak^†††^ include monitoring for infections in exposed persons, conducting syndromic and laboratory surveillance, planning for epidemiologic investigations, and assessing performance of existing medical countermeasures including diagnostic tests, vaccines, and therapeutics. To assess A(H5N1) virus pathogenesis, severity, and transmissibility in an animal model of infection, CDC is also conducting laboratory experiments in ferrets.

This activity was reviewed by CDC, deemed not research, and was conducted consistent with applicable federal law and CDC policy.^§§§^ Ferret studies were approved by the CDC Institutional Animal Care and Use Committee.

### Monitoring of Persons Exposed to Influenza A(H5) Viruses

In 2014, CDC began monitoring persons exposed to infected poultry when HPAI A(H5) viruses were first detected in poultry and wild birds in North America (*5*). Recommendations are to monitor persons exposed to infected birds, poultry, or other animals for 10 days after their last exposure and to test symptomatic persons for influenza A viruses by RT-PCR assay using H5-specific primers and probes, in coordination with state or local health departments (*6*).

During February 2022–May 2024, approximately 9,400 persons in 52 jurisdictions have been monitored. As of May 22, 2024, approximately 350 farm workers had been or were currently being monitored for illness after exposure to infected cows or infected raw cow’s milk; the number of persons monitored continues to increase; data are updated weekly.^¶¶¶^ Monitoring is performed either through direct daily contact by state or local health departments or by providing persons with information on how to self-monitor and where to seek testing and possible treatment should they experience symptoms. The most recent human A(H5) case was identified through active, daily monitoring of exposed farm workers using a text-based illness monitoring program in Michigan (*7*).

### National Surveillance Activities

CDC’s influenza surveillance systems**** collect information to track trends in influenza activity and detect changes in circulating influenza viruses, including detection of novel influenza A viruses year-round. Human cases of novel influenza A virus infection have been nationally notifiable since 2007; every identified case is investigated and reported to CDC.

Through approximately 300 clinical laboratories, CDC monitors changes in the percentage of influenza tests with positive results in clinical settings. The National Syndromic Surveillance Program collects data from emergency departments and other health care settings, facilitating the detection of unusual trends in influenza diagnoses, including in jurisdictions where A(H5N1) viruses have been identified in animals.

CDC’s National Wastewater Surveillance System^††††^ complements other existing human influenza surveillance systems in monitoring influenza trends. These monitoring methods detect influenza A viruses but do not distinguish subtypes of influenza A, meaning that current wastewater testing can detect A(H5N1) viruses but cannot distinguish them from other influenza A viruses or determine the source of the influenza A viruses (e.g., humans versus animals or animal products). Together, these systems provide visibility into U.S. influenza activity. As of May 18, 2024, no indicators of unusual human influenza activity, including A(H5N1 virus), had been detected in humans through these systems.

CDC’s molecular diagnostic assays are used at more than 100 public health laboratories in all 50 states and other U.S. jurisdictions to detect seasonal and novel influenza A viruses; nine centers also perform genetic sequencing for virus characterization. Statistical methods are used to determine the number of specimens needed to have 95% confidence that at least one novel influenza A virus among all influenza positive specimens per week would be detected given varying influenza prevalence; the number varies by timing during the season. Each state’s contribution is proportional to its population and has been set as a national weekly goal for public health laboratory testing.^§§§§^

### Spring and Summer Activities

Multiple efforts are underway to enhance influenza surveillance activities through the spring and summer as part of this response. CDC is working with commercial laboratories to increase submission of influenza-positive test specimens to public health laboratories to increase the number of specimens available for virus subtyping. Approximately 140,000 of these H5-specific tests are already prepositioned at the state and local level, and another 750,000 tests are available for distribution if needed. CDC also continues to collaborate with manufacturers of commercial diagnostic tests with the goal of having an A(H5N1) test that is widely available if needed. Surveillance for laboratory-confirmed, influenza-associated hospitalizations will also continue during the spring and summer through the Influenza Hospitalization Surveillance Network (FluSurv-NET), which typically conducts surveillance during October 1–April 30 of each influenza season. As well, CDC is working with state and local public health partners, with outreach to providers and clinics, to increase awareness about A(H5N1) so that influenza is considered in patients with conjunctivitis or respiratory illness after exposures, including agricultural fair attendance, that might increase the risk of novel influenza A virus infection. 

### Medical Countermeasures

As a World Health Organization Collaborating Center, and in partnership with the Administration for Strategic Preparedness and Response (ASPR), CDC regularly develops novel influenza A candidate vaccine viruses (CVVs) for pandemic preparedness. Antigenic characterization of the A(H5N1) virus isolated from the Texas farm worker (A/Texas/37/2024) with ferret antisera produced against existing CVVs confirmed two clade 2.3.4.4b A(H5) CVVs have good cross-reactivity to this virus. Under the National Pre-Pandemic Influenza Vaccine Stockpile (NPIVS) program, ASPR has shared these CVVs with Food and Drug Administration (FDA)–licensed pandemic influenza vaccine manufacturers and has completed initial production of bulk antigen. ASPR is also supporting clinical evaluation of safety and immunogenicity of vaccines using antigen manufactured from one of these CVVs, influenza A/Astrakhan/3212/2020–like virus vaccine, in combination with different adjuvants that are stockpiled under the NPIVS. The clinical study (NCT05874713)^¶¶¶¶^ testing cell-based antigen combined with MF59 adjuvant, according to the AUDENZ-licensed manufacturing process, has completed enrollment. The egg-based antigen, produced according to the Q-PAN-licensed process, combined with AS03 adjuvant clinical study (NCT05975840)***** is also fully enrolled. ASPR is planning additional clinical studies for combining egg-based antigen with both AS03 and MF59 adjuvants with enrollment expected to start in late summer 2024. If needed, and dependent upon FDA review and regulatory action allowing use, these vaccines could be the first allotment of vaccines used while additional manufacturing, starting with the stockpiled antigens and adjuvants, ramps up for full-scale production.

Four FDA-approved antiviral drugs (baloxavir marboxil, oseltamivir, peramivir, and zanamivir) are recommended for influenza treatment in the United States.^†††††^ CDC has conducted phenotypic testing of antiviral susceptibility and found that the A(H5N1) virus isolated from the Texas farm worker is susceptible to baloxavir marboxil (Xofluza, Genentech) and to neuraminidase inhibitors, including oseltamivir (generic or Tamiflu, Genentech). Oral oseltamivir treatment is recommended for persons with confirmed or suspected A(H5N1) virus infection.^§§§§§^ Oral oseltamivir is also recommended for postexposure prophylaxis (using twice daily treatment dosing) of close contacts (e.g., household members) of a confirmed A(H5N1) case. Observational studies of patients infected with older and different clades of A(H5N1) viruses, (i.e., not the current clade 2.3.4.4b viruses identified in the United States) have found that starting oseltamivir treatment within 2 days of symptom onset was significantly associated with survival benefit compared with no treatment or later initiation of oseltamivir treatment after symptom onset (*8**,**9*). All four antivirals are available in the Strategic National Stockpile and in many state-managed stockpiles, both of which can be deployed to assist with supply chain constraints should they arise.

The National Institute of Allergy and Infectious Diseases (NIAID) continues to investigate the efficacy of novel direct-acting antiviral medications and host-targeted molecules as well as broadly neutralizing antibodies and more targeted monoclonal antibodies aimed at A(H5N1) viral-specific surface antigens that could protect from death or severe respiratory disease. 

### Epidemiologic Investigations 

To better ascertain and define the risk to humans, CDC is working with states to plan epidemiologic investigations in collaboration with affected farms and health and agricultural partners at local, state, and federal levels. Important public health questions might be addressed through in-depth studies with specimen collection and surveys (Box). CDC conducted a similar study in response to poultry outbreaks of A(H5N1) in 2022 (*10*).

BOX **Key epidemiologic questions to define the risk of highly pathogenic avian influenza A(H5N1) viruses to humans and to guide evidence-based recommendations — United States, 2024**

1. Is there evidence of influenza A(H5N1) virus infections in human populations?

2. If human illness is identified, what is the clinical spectrum of illness?

3. What are the rates of asymptomatic human infection with influenza A(H5N1) virus?

4. What are the routes of exposure to influenza A(H5N1) virus on farms and dairies, and what is the risk for zoonotic transmission?

5. What behaviors, including use of personal protective equipment, are associated with human infection or protection from infection with influenza A(H5N1) virus?

## Discussion

CDC is collaborating with the U.S. Department of Agriculture, FDA, ASPR, the Health Resources and Services Administration, NIAID, and state and local public health and animal health officials using a coordinated One Health approach to identify and prepare for developments that could increase the risk to human health. Substantial challenges to identifying and interviewing persons exposed to cattle infected with A(H5N1) viruses for illness monitoring or epidemiologic studies exist. Workers exposed to A(H5N1) viruses might represent socioeconomically vulnerable, or otherwise hard-to-reach populations, including those who live in rural or remote areas; or they might be migrant, transient, or undocumented workers. Further, persons might not be aware of the risks or potential signs and symptoms associated with exposure; dairy farmers and the dairy industry have not previously been major partners in outreach about avian influenza. Recommendations for worker protection have been recently updated^¶¶¶¶¶^ and disseminated.

Once exposed persons are identified, defining exposure periods is also difficult. A(H5N1) disease is widespread in poultry, and mortality is high. Rapid depopulation of affected flocks facilitates monitoring of exposed workers because it creates a finite 10-day monitoring window after exposure. In contrast, illness in cows can last for 2–4 weeks, and the duration of infectious virus shedding in cows is unknown. In addition, A(H5N1) virus infection has been identified in some cows without signs of illness; thus, some workers might be unaware of their exposure. Recent testing did not detect live, infectious A(H5N1) viruses in retail dairy samples; however, identification of A(H5N1) viral fragments in approximately one in five retail milk samples from across the country (*4*) suggests that A(H5N1) virus infections of cattle might be widespread. Therefore, monitoring of exposed or potentially exposed persons and animals might be protracted and resource-intensive.

Interpretation of surveillance data can be challenging given that A(H5N1) virus infections might manifest signs and symptoms similar to those associated with infections caused by other pathogens. During periods of low U.S. influenza virus circulation (e.g., spring and summer), syndromic and wastewater surveillance might more readily identify unusual signals in influenza-related symptoms or activity. However, using these systems to detect novel influenza A virus infection trends in the fall and winter, once seasonal influenza A virus circulation increases, will likely be complicated. Interpretation of wastewater data are further limited by the inability to distinguish between human and animal source material.

Currently circulating A(H5N1) viruses do not have the ability to easily bind to receptors that are most prevalent in the human upper respiratory tract and therefore are not easily transmissible to and between humans (*2*). However, because of the widespread global prevalence of A(H5N1) viruses in birds and other animals, continued sporadic human infections are anticipated. Further, if a novel influenza A virus acquires the ability to infect and be transmitted easily between persons in a sustained manner, an influenza pandemic could occur. Thus, investigation of every novel influenza A virus case in humans and comprehensive worldwide surveillance is critical to public health preparedness efforts.

### Implications for Public Health Practice

CDC considers the current health risk to the U.S. public from A(H5N1) viruses to be low. However, persons who have job-related or recreational exposure to infected birds, poultry, dairy cattle, or other infected animals or contaminated materials, including raw cow’s milk, are at increased risk for infection; these persons should take appropriate precautions, including using recommended personal protective equipment, self-monitoring for illness symptoms (*6*), and seeking prompt medical evaluation if they are symptomatic, including influenza testing and antiviral treatment if indicated. FDA has confirmed that pasteurization inactivates A(H5N1) viruses, and that the commercial milk supply is safe for consumption (*4*); however, all persons should avoid consuming raw milk or products produced from raw milk. A coordinated and comprehensive One Health response to this ongoing outbreak of A(H5N1) virus infections in dairy cows, poultry, and other animals is needed to identify and prepare for any developments that indicate an increase in the risk to public health.

## Footnotes

*
https://www.dshs.texas.gov/news-alerts/health-alert-first-case-novel-influenza-h5n1-texas-march-2024#:~:text=Summary,patient%27s%20primary%20symptom%20was%20conjunctivitis

†
https://emergency.cdc.gov/han/2024/han00506.asp

§
https://www.aphis.usda.gov/news/agency-announcements/federal-state-veterinary-public-health-agencies-share-update-hpai

¶
https://wahis.woah.org/#/in-review/4451?fromPage=event-dashboard-url

**
https://www.cdc.gov/flu/avianflu/spotlights/2023-2024/h5n1-analysis-texas.htm

††
https://www.michigan.gov/mdhhs/inside-mdhhs/newsroom/2024/05/22/influenza-a-detection

§§
https://www.aphis.usda.gov/livestock-poultry-disease/avian/avian-influenza/hpai-detections/livestock

¶¶
https://www.cdc.gov/media/releases/2022/s0428-avian-flu.html

***
https://www.cdc.gov/one-health/about/index.html

†††
https://www.cdc.gov/flu/avianflu/what-cdc-doing-h5n1.htm

§§§ 45 C.F.R. part 46.102(l)(2), 21 C.F.R. part 56; 42 U.S.C. Sect. 241(d); 5 U.S.C. Sect. 552a; 44 U.S.C. Sect. 3501 et seq.

¶¶¶
https://www.cdc.gov/flu/avianflu/h5-monitoring.html

****
https://www.cdc.gov/flu/weekly/index.htm

†††† https://www.cdc.gov/nwss/wastewater-surveillance/Flu-A-data.html

§§§§
https://www.aphl.org/aboutAPHL/publications/Documents/ID-Influenza-Right-Size-Roadmap-Edition2.pdf

¶¶¶¶
https://www.clinicaltrials.gov/study/NCT05874713?term=NCT05874713&rank=1

*****
https://www.clinicaltrials.gov/study/NCT05975840?term=NCT05975840&rank=1

†††††
https://www.cdc.gov/flu/professionals/antivirals/index.htm

§§§§§
https://www.cdc.gov/flu/avianflu/novel-av-treatment-guidance.htm

¶¶¶¶¶
https://www.cdc.gov/flu/avianflu/h5/worker-protection-ppe.htm
